# Traditional herbal medicine in Far-west Nepal: a pharmacological appraisal

**DOI:** 10.1186/1746-4269-6-35

**Published:** 2010-12-13

**Authors:** Ripu M Kunwar, Keshab P Shrestha, Rainer W Bussmann

**Affiliations:** 1 Ethnobotanical Society of Nepal, GPO Box 5220, Kathmandu, Nepal; 2 Natural History Museum, Swayambhu, Tribhuvan University, Kathmandu, Nepal; 3 William L. Brown Center, Missouri Botanical Garden, St. Louis, MO 63166-0299, USA

## Abstract

**Background:**

Plant species have long been used as principal ingredients of traditional medicine in far-west Nepal. The medicinal plants with ethnomedicinal values are currently being screened for their therapeutic potential but their data and information are inadequately compared and analyzed with the *Ayurveda *and the phytochemical findings.

**Methods:**

The present study evaluated ethnomedicinal plants and their uses following literature review, comparison, field observations, and analysis. Comparison was made against earlier standard literature of medicinal plants and ethnomedicine of the same area, the common uses of the *Ayurveda *and the latest common phytochemical findings. The field study for primary data collection was carried out from 2006-2008.

**Results:**

The herbal medicine in far-west Nepal is the basis of treatment of most illness through traditional knowledge. The medicine is made available via ancient, natural health care practices such as tribal lore, home herbal remedy, and the *Baidhya*, *Ayurveda *and *Amchi *systems. The traditional herbal medicine has not only survived but also thrived in the trans-cultural environment with its intermixture of ethnic traditions and beliefs. The present assessment showed that traditional herbal medicine has flourished in rural areas where modern medicine is parsimoniously accessed because of the high cost and long travel time to health center. Of the 48 Nepalese medicinal plants assessed in the present communication, about half of the species showed affinity with the common uses of the *Ayurveda*, earlier studies and the latest phytochemical findings. The folk uses of *Acacia catechu *for cold and cough, *Aconitum spicatum *as an analgesic, *Aesculus indica *for joint pain, *Andrographis paniculata *for fever, *Anisomeles indica *for urinary affections, *Azadirachta indica *for fever, *Euphorbia hirta *for asthma, *Taxus wallichiana *for tumor control, and *Tinospora sinensis *for diabetes are consistent with the latest pharmacological findings, common Ayurvedic and earlier uses.

**Conclusions:**

Although traditional herbal medicine is only a primary means of health care in far-west Nepal, the medicine has been pursued indigenously with complementing pharmacology and the *Ayurveda*. Therefore, further pharmacological evaluation of traditional herbal medicine deserves more attention.

## Background

Current estimates suggest that, in many developing countries, about two thirds of the population relies heavily on traditional practitioners and medicinal plants to meet primary health care needs [1]. Although modern medicine may be available in these countries, traditional herbal medicine is often been used for historical, cultural, and ecological reasons, in particular this is due to continued availability [2], better compatibility [3] and high acceptance [4]. Traditional herbal medicine possesses greater significance in Nepal Himalaya hence interest in herbal medicine has gradually increased in recent years [5]. As a result, the medicine all over the world is nowadays revalued by extensive researches on base materials plant species and their therapeutic principles, however to date only about five percent of the total plant species have been thoroughly investigated [6-8] to ascertain safety and efficacy of traditional medicines.

Plant species have long been the principal ingredients of traditional medicine [9] and their use dates back to the beginning of human civilization [10]. Herbal medicine has clearly recognizable therapeutic effects [11] as well as some toxic side-effects [12]. Thus, Nepalese medicinal plants with ethnomedicinal properties are being screened for their active pharmacological effects [13]. The present study therefore evaluated the ethnomedicinal uses of the selected 48 second priority medicinal plants of Baitadi, Dadeldhura and Darchula districts of far-west Nepal and comparatively assessed their uses against earlier standard literature on medicinal plants of the same area, the common uses of the *Ayurveda *(an ancient traditional system of herbal medicine in the Himalaya) and the latest phytochemical findings.

## Materials and methods

**Figure 1:**
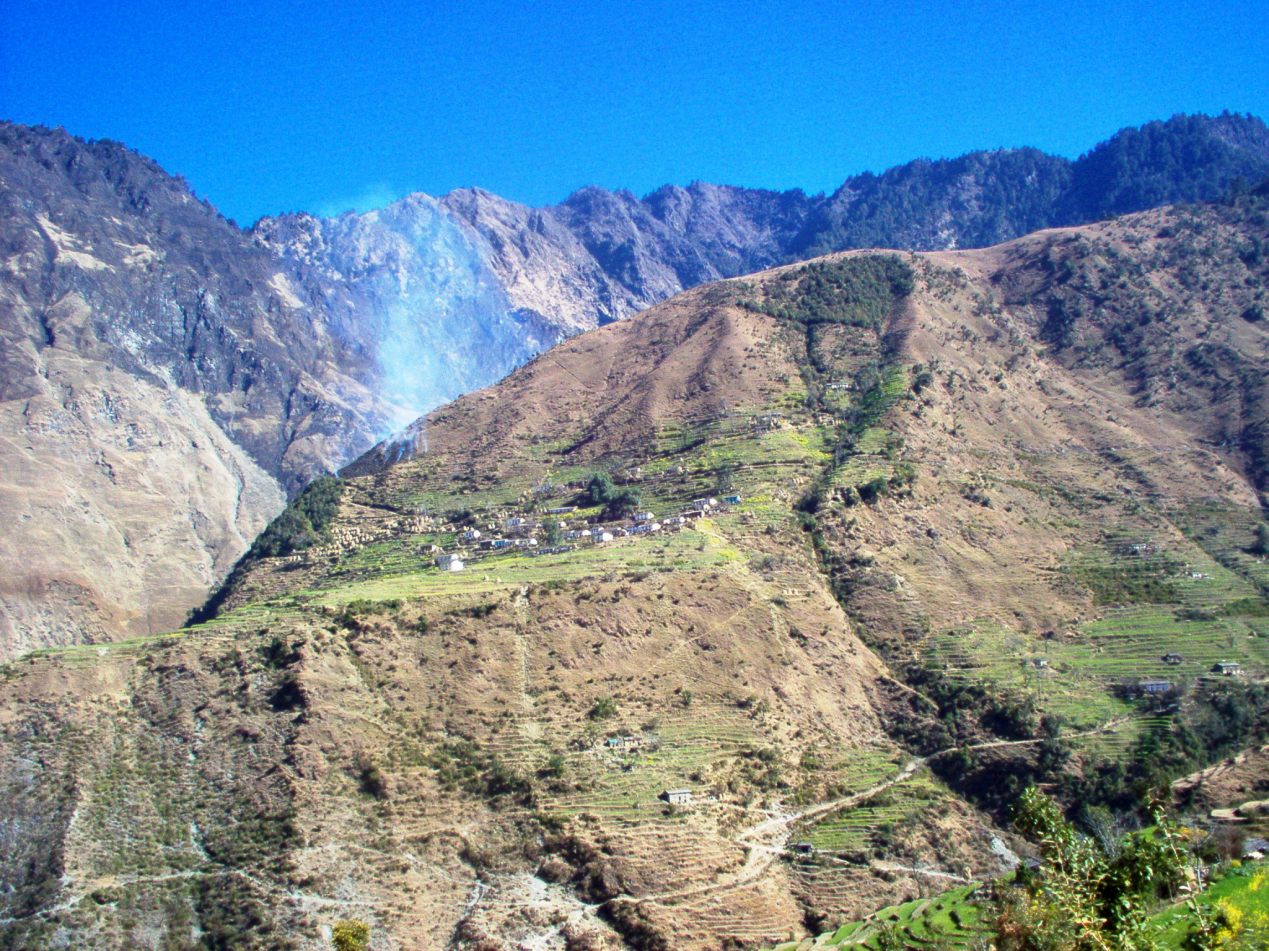
**Study site: Khar VDC, Darchula district**.

The field study for primary data collection was carried out in the Baitadi, Dadeldhura, and Darchula districts of far-west Nepal from 2006-2008. The districts stretch between 29°01' and 30°15'N latitude, 80°03' and 81°09'E longitude and 357 m - 7132 m altitude. The study sites were Anarkholi, Dasharathchand, Jhulaghat, Khodpe, Kulau, Pancheswor, Patan, Salena, and Sera (Baitadi); Brikham, Jakh, Jogbudha, Patram, and Rupal (Dadeldhura), and Dumling, Gokule, Joljibi, Khalanga, Khar (Figure 1), Lali, and Uku (Darchula). All three districts are situated along the western borders of the country and lie adjacent to India. Due to variations in altitude, topography, and bio-climate within the districts, the diversity of medicinal plants and knowledge of utilization are vast. The subsistence use is profound particularly for home herbal healing [5,14]. There are a number of diverse ethnic groups in the area. The largest ethnic group is the Chhetri (more than 50%), followed by Brahmin (about 20%), *Dalits *(about 10%), Thakuri (7%), Magar (2 %), and a few other groups. The first two groups are considered privileged and the rest are considered ethnic (*Janajati*) and disadvantaged (*Dalits*). Ethnic and disadvantaged groups have easy access opportunities provided by the government.

### Field surveys and data collection

Four field surveys were carried out during different seasons of the year (May, December 2006, February 2007, March-April 2008). Each survey lasted over 20 days in the field. Primary data collection, after establishing oral informed consent with the participating communities, consisted of group discussions, informal meetings, schedule surveys, key informant surveys, cross-checking, and field observations. In all surveys, four group discussions and six informal meetings were held; in total 172 individuals were consulted. Informal meetings were held in villages while staying with them. The traditional healers (*Baidhyas*) and women representing major ethnic groups, castes, and occupations were encouraged to participate. *Baidhyas *are traditional medicinal practitioners particularly of the western Nepal mid-hills [15] and adjoining areas of India [16]. Women were active participants of the informal meetings. Among the respondents, 3% were traditional healers, 12% were ethnic groups, and 21% were women.

All plant species encountered during field observations were recorded. Medicinal plant species were collected during the day and displayed during evening meetings for discussion. Both the collections and surveys/discussions were facilitated by local assistants, and the information was sought about vernacular dialects, indigenous uses of the species and participants' priority on species. Ranking was followed to categorize the first, second and third priority medicinal plant species. The species enumerated in the present study were the second priority medicinal plants of the local communities with informant consensus factor less than 0.85. The first priority medicinal plants with quantitative ethnomedicine were already discussed [14,17].

Matching information from at least three respondents (mentions) was counted as a common response for the analysis. The single most common folk use of each species was valued for further discussion. Common species and mono specific genera which were well known by their dialect names were used only for discussion and not managed as voucher specimen for further identification. Voucher specimens were collected, and vernacular names and folk uses were recorded for each specimen. Specimen collection was made following Cunningham [18], and plants were identified to species level. Most of the species were identified in the field using literature [19,20]. The remaining unidentified species were identified and housed in Kathmandu at Tribhuvan University Central Herbarium (TUCH), Department of Botany, Tribhuvan University, Nepal.

The observations of the present survey were compared to earlier observations, latest common phytochemical findings and common uses of the *Ayurv*eda. The common uses of the *Ayurveda *were taken from the following literature [21-27]. Literature [28-33] of Nepal were used as reference for earlier ethnomedicinal information of the same area. Pharmacological information was retrieved from internet sources (available till June, 2010) and relevant journals; most of them were accessed from USA. About 240 research papers and articles were reviewed for analysis.

## Results and Discussion

### Traditional herbal medicine

Traditional herbal medicine has been used since ancient time in many parts of the world where access to formal and modern healthcare is limited. Nepal is not exempt and in mid-hills, mountainous and rural areas of the country where access and services are limited, herbal medicine is the basis of treatment of most illness through traditional knowledge. It is estimated that approximately 90% of the Nepalese people reside in rural areas where access to government health care facilities is lacking [30]. These people rely predominantly on traditional herbal medicine. Traditional medicine is made available via ancient, natural health care practices such as tribal lore, home herbal remedy, and the *Baidhya*, *Ayurveda *and *Amchi *(traditional healing system of Tibet and mountain areas of Nepal) systems. The former one is innate to the tribal group (i.e. *Raute *in study area) [34]. Home herbal remedy and the *Baidhya *system are indigenous to far-west Nepal [14,15] and are partly influenced by the *Ayurveda *[35]. Extant of home herbal remedy in far-west Nepal is also due to relatively homogenous resource users and less encroachment from immigrants. Home herbal remedy and *Baidhya *system, yet transformations of the *Ayurveda*, are well established and practiced in the study area. The *Amchi *system is widely accepted and practiced throughout high altitude areas of Nepal [10] and is important in Darchula district, albeit with some modifications [29].

As communicated by Kunwar *et al*. [17], the knowledge base for traditional herbal medicine stems from spirituality, customs, livelihood strategies and available nearby resources. Medicinal herbs are main ingredients of traditional herbal medicine, and the traditional herbal medicine is considered as the main lifeline [36], the first choice [37], fewer side-effects, better patience tolerance, relatively less expense, and cultural acceptance and long history of use, in comparison to western medicine. Thus, the traditional herbal medicine has not only survived but also thrived in the trans-cultural environment with its intermixture of ethnic traditions and beliefs. Most of the time, this knowledge is passed on orally and therefore is endangered. Particularly the *Amchi *knowledge is passed down through dedicated apprenticeships under the tutelage of senior *Amchi *[38]. Although traditional herbal medicine is effective in treatment of various ailments with considering ritual and socio-cultural customs [39], very often the medicine is used indigenously with indifference to the scientific knowledge and their possible side effects were overlooked. The dearth of reports of adverse effects and interactions probably reflect a combination of under-reporting and the benign nature of most herbs used [40]. Therefore, the traditional herbal medicine deserves a great scope of research in the light of modern science.

The present assessment showed that traditional herbal medicine has flourished in rural areas where modern medicine is parsimoniously accessed as a result of the high cost and long travel time to health center. Moreover inadequate modern medical resources/facilities and government subsidies also made traditional herbal medicine pertinent in Nepal. It is estimated that there is one physician for every 20,000 people whereas there is more than one healer for every 100 people in Nepal [41,42]. Herbal medicine prescribed by healers is either preparation based on single plant part or a combination of several plant parts. However, we dealt only the primary one for further discussion in the present study. Many of the plants most often used in study area to treat ailments are also commonly used all over Nepal. Particularly the ethnic groups and scheduled caste are the major stakeholders of the traditional herbal medicine [43], so, traditional medicine is still the mainstay of health care in the rural areas of Nepal where the majorities of the denizens are from ethnic groups and scheduled castes.

### Medicinal plants and their uses

Of the 48 species from 46 genera and 40 families (Table 1) discussed in the present study, indigenous uses of about 70% species resembled to the earlier ethnomedicinal reports. The indigenous uses of about 50% species had affinity to the *Ayurveda*, and about 40% species were found to have efficacy in pharmacology. Fabaceae, Moraceae and Rosaceae were represented by the greatest number of species (3 each), followed by Euphorbiaceae and Lamiaceae (2 each) for herbal medicine in study area. A total of 30 ailments were reported in the present study, and among these inflammation, cuts & wounds, diarrhea & dysentery and fever were considered as common, and the maximum number of medicinal plant species were used against, six species to each category and four for the latter. Similar observation of maximum number of species used for fever and cuts & wounds was reported by Manandhar [34]. The plant parts used for herbal remedies were bark, flower, fruit, leaf, milk/latex, root/rhizome, seed, shoot, wood, and the whole plant. Plant parts root/rhizome, leaf, and fruits, etc. were most frequently utilized.

**Table 1:** Major uses of the medicinal plants, their chemical constituents, and latest common pharmacological findings (species are in order of references)

SN	Scientific name, local name, family and voucher code	Folk use found in present survey	Major folk uses in previous studies	Major uses in the *Ayurveda*	Selected major chemical constituents	Latest common pharmacological findings
1.	*•⊗Lobelia pyramidalis *Wall. **Campanulaceae **Lobelia (E), Aklebir (N), Eklebir (S), 569/00. Syn. *L. nicotianaefolia *Roth	Juice of leaves and flowers is rubbed on body parts during body ache.	Leaves and inflorescence are antispasmodic [30] and used for asthma, bronchitis and fever [31].	Leaves and flowers are antispasmodic and they are used as an expectorant. Plant is used for sciatia and back pain [21].	Lobeline, radicamine.	Lobeline may cause nausea, vomiting and diarrhea [38].

2.	♥*⊗Cannabis sativa *L. **Cannabaceae **Hemp (E), Ganja (N), Bhang (S). Syn. *C. indica *Lam.	Leaf juice is applied to control bleeding.	Leaf juice is useful for healing wounds, control bleeding and stomachache [32].	Plant is efficacious for diarrhea. It is also used as antispasmodic [21] and sedative [25].	Cannabigerol, cannabidiol, friedelin, lectins [32].	Leaves are used as snuff for smoking and are given internally to relieve pain and swelling [27]. Lectins possess haema-gluttinating properties [38].

3.	*⊗Scutellaria discolor *Colebr. **Lamiaceae **Ratpatya (L), Dampate (N) KU 07263. Syn. *S. indica *Blume	Whole plant and leaf paste is useful for cuts and wounds.	Plant juice is useful for headache and fever [28] and wounds healing [30].	Plant juice is used for rheumatism [147].	Wogonin	Root juice is given in indigestion and wogonin exerts anxiolytic effects [135]. Plant and root extract is used for rheumatism [136].

4.	*⊗Ficus palmata *Forssk. **Moraceae **Bedu (N). Syn. *F. virgata *Wall.	Plant milk is useful for taking out the thorns from wounds.	Plant latex is used to expel the spines [30]. Fruits are used for constipation, lungs and bladders diseases [33].	Fruits are taken for lungs disorders [147].	Friedelin, tannins.	Fruits act as demulcent and laxative and are useful for lungs, spleen and bladders [136].

5.	*♥⊗Grewia disperma *Rottb. **Tiliaceae **Viywal (L), Syalpuchre (N). Syn. *G. serrulata *DC.	Root juice is taken as expectorant. Wood paste is applied for skin diseases (no other information given).	Root juice is taken during cough and cold. Bark paste is expectorant and used for boils [33].	Root juice is used for controlling bleeding and bronchitis [147].	--	Plant is applied in bleedings and bronchitis. Fruits are valued as cardiotonic [136].

6.	⊗*Podophyllum hexandrum *Royle **Berberidaceae **Podophyllum, May apple (E), Laghupatra (N), Hatkaudo (L), Hansapadi, Laghupatra (S), 583/00. Syn. *P. emodi *Wall. ex Hook. f. & Thomson	Root juice is taken for liver complaints (no other information given).	Plant is hepato-stimulant and purgative [15,31]. Root paste is applied on ulcer, cuts and wounds [32].	Root extract is purgative [147].	Aryltetralin, astragalin, lignan, picropodophyllin, podophyllotoxin, quercetin [27].	Plant lignan is hepatotoxic [62], aryltetralin is antifungal [148], and podophyllotoxin is antitumour. Aqueous extract of plant has antitumor effects [149].

7.	•⊗*Potentilla fulgens *Wall. Ex Hook. **Rosaceae **Himalayan Cinquefoil (E), Phosre (L), Bajradanti (N), Kanthamun (S), 93/00. Syn. *P. siemersiana *Lehm.	Dried roots are eaten as dentifrice.	Root used as tooth powder for toothache [30,31].	Root powder is used for toothache [25].	Carotene, coumarins, flavonoids, polyphenols, sterols [25].	Aqueous extract of the plant reduced germination of food crops [150].

8.	♥*⊗Carum carvi *L. **Apiaceae **Caraway (E), Jangali jira (L), Kalo jira (N). Syn. *Apium carvi *L.	Fruits are applied against swelling of breast and testicles.	Plant fruit juice is useful for muscular swellings [30]. Raw fruits are stomachic and carminative [31].	Plant seeds are useful in uterinal complaints [22], and used as antidysenteric, astringent, anthelminthic and carminative [151].	Camphene, carvone, caryophyllene, limonene, myrcene, pinene, sabinene, scopoletin, umbelliferone [100].	Fruits are good for painful swelling [152]. Carvone is anthelmintic [153] and antioxidative [154]. Essential oil is antibacterial [155] and antitumeric [156]. Aquous fruit extract is used against hypertension, gonorrhoea [157] and diabetes [158].

9.	♥*•⊗Aconitum spicatum *(Bruhl) Stapf. **Ranunculaceae **Nepalese Aconite (E), Bikh (N), Bish (S), KU 07233. Syn. *A. ferox *var. *spicata *Bruhl	Root juice is antipyretic and analgesic.	Tubers are used after detoxification [31] as antipyretic and analgesic [32].	Plant tuber is antipyretic and analgesic [25]. Plant root is used for tonsillitis, sore throat, gastritis, and debility [152].	Bikhaconitine, caffeic acid, diterpenoids, lupenoic acid, pseudaconitine.	Caffeic acid of *Aconitum species *is antioxidative and anti-inflammatory [138].

10.	♥•⊗*Taxus wallichiana *(Zucc.) Pilger **Taxaceae **Himalayan Yew (E), Kandeloto (L), Lothsalla (N), Madhuparni (S), 99/00. Syn. *T. baccata *auct. non.	Leaf juice is used for cancer and bronchitis.	Bark and leaf juice is useful for asthma, bronchitis and cancer [30,32].	Dried leaves are considered to be useful for asthma, bronchitis, hiccough, epilepsy, diarrhea and headache [151].	Abeotaxane, baccatin, cephalomannin, docetaxol, paclitaxel, taxol [159].	Fractions of extract of leaves inhibited pregnancy in 60% female rats [22]. It cures vitiation of blood [100] and inhibits tumor growth [101].

11.	♥*•⊗Acacia catechu *(L.f.) Willd. **Fabaceae **Cutch tree (E), Khair (N), Khadirah (S). Syn. *A. catechoides *(Roxb.)	Wood is used as local tea for cough and cold.	Wood decoction is applied on nosebleeds, skin eruptions and toothache [30] and for cough and bodyache [32].	Plant decoction is used for skin diseases and mouth and mucous defects [21]. Wood is useful for cough and diarrhea [25].	Acacatechin, afzelchin, catechuic acid, catechutannic acid, cyanidanol, dimeric procyanidine, epicatechin, isorhamnetin, phlebotanin, quercetin, taxifolin, tryptamine, vernolic acid [160].	Cyanidanol, an active ingrediant of *Acacia catechu*, is claimed to be effective for treating liver diseases [95]. Catechu has hypoglycaemic [161], antipyretic [162] and digestive properties [163]. Taxifolin has antioxidant and anti-inflammatory activities [164]. Catechuic acid is valued for expectoration for chest infection [165].

12.	⊗*Engelhardia spicata *Leschen. ex Blume **Juglandaceae **Mahuwa (N). Syn. *E. colebrookeana *Lindl. ex Wall.	Flower juice is drunk for abdominal pain.	Flower juice is useful for abdominal pain [5], cough and cold [166].	Bark is used as piscidal [147].	Engelhardtione, oleanolic acid.	Engelhardtione possesses antituberculer activities [167].

13.	•*Spondias pinnata *(L.f.) Kurtz **Anacardiaceae **Bile tree, Wild mango (E), Amaro (L), Pitavraksha (S). Syn. *S. mangifera *Willd.	Plant latex is applied for wounds and cuts.	Plant juice is useful for dysentery and rheumatism [30] plant latex is used for bilious dyspepsia [33].	Latex is demulcent [27].	Alanine, amyrin, cystine, lignoceric acid, oleanolic acid, serine [27,100].	Flavonoids of the plant have been known to inhibit intestinal motility and hydroelectrolytic secretion, which are known to be altered for diarrhoeal conditions [168].

14.	⊗*Schleichera oleosa *(Lour.) Oken **Sapindaceae **Macassar tree, Honey tree, Lac host tree (E), Kusum (N). Syn. *S. trijuga *Willd	Fruits are eaten as an anthelmintic.	Fruits are used for heat stroke, and valued as appetite stimulant [30], anthelmintic and tonic [33].	Seed oil is used for skin diseases [27].	Behemin, campesterol, gadoleic acid, oleic acid, oxalic acid, palmitic acid, stearic acid, tartaric acid [27].	Fruit juice stimulates hair growth [169].

15.	*Rhododendron campanulatum *D.Don **Ericaceae **Chimal (N) 89/00. Syn. *R. wallichii *Hook.f.	Flowers are used in body ache and throat pain. Seeds aid digestion.	Flowers are useful for skin diseases [33].	Leaf extract is used for rheumatism and syphilis [147].	Amyrin, andromedotoxin, campanulin, chlorogenic acid, epifriedelinol, gallic acid, phenols, quercetin, ursolic acid [170,171].	Plant andromedotoxin is poisonous to the livestock [136]. Good amount of phenols and ursolic acid in the plant help to reduce risk of cardiovascular diseases [129,171] and cancer [172,173].

16.	*♥Boehmeria platyplylla *D.Don **Urticaceae **Chinese grass (E), Kamle (L), Gargalo (N). Syn. *B. macrostachya *Wedd.	Root paste is applied on control bleeding.	Root juice is given for stomachache [28] and dysentery [30].	Plant juice is poisonous to fish [147].	Acetophenone, cryptopleurine, secophenanthroqlinolizidine [174].	Leaf juice is applied on cuts and wounds [174].

17.	♥•⊗*Andrographis paniculata *(Burm. f.) Wall. ex Nees **Acanthaceae **Creat (E), Kitatikta, Kalmegh (N), Bhunimbah (S). Syn. *A. subspathulata *Clarke.	Raw plant root juice is considered as antipyretic and effective against infections.	Plant is useful for curing malarial and intermittent fever, dysentery and liver disorders [32].	Plant is effective for dermatologial diseases [27]. It is useful in malarial and intermittent fevers [175].	Andrographolide, caffeic acid, kalmeghin, neoandrographolide, panicolide.	Plant is immunostimulant [58], anti-inflammatory [53], antibacterial [59], analgesic [60] and antiprotozoal [61]. Kalmeghin increases biliary flow and liver weight [175] and aids intestinal digestion [176] and liver protection [177,178].

18.	•*Sapium insigne *(Royle) Benth. ex. Hook. f. **Euphorbiaceae **Tallow tree (E), Khirro (N).	Milky latex is skin irritant and sprayed as fish poison in stream and tributaries.	Bark latex is used to dispel works and germs for livestock [33].	Latex is vesicant [147].	Corilagin, guijaverin, nicotiflorin, phorbol esters, quinic acid, rutin, scopolin [179].	Leaf extract is used for snake bite [180].

19.	*⊗Vitex negundo *L. **Verbenaceae **Negunda Chaste tree (E), Simali (N), Nirgundhi (L), Shephali (S). Syn. *V. cannabilifolia *Sieb. & Zucc.	Leaf juice is useful in stomachache.	Plant juice is used for headache [28]. Leaf juice is useful for gastric troubles [30] and used for common cold, fever and dermatitis [31].	Plant is used for fever and nerve defects [21].	Agnusid, aucubin, casticin, hentriacontane, luteolin nishidine, peduncularisid, vanilic acid, vitexin [100,181].	Leaf extract shows antibacterial [103] and weak antifungal properties [104] and it is good for lowering blood glucose levels [105], cancer treatment [106] and acne control [107]. It is useful for inhibition of edema [108,109] and tracheal contraction [110].

20.	♥*⊗Skimmia anquetilia *N.P. Taylor & Airy Shaw **Rutaceae **Chillo pate (L), Narpati (N).	Leaf infusion is taken for headache and for freshness.	Leaves are aromatic and used for headache and general fever [15,33].	--	Linalool, geraniol, pinene, scopoletin, skimmianine, umbelliferone [181,182].	Linalool could possess anxiolytic effect [137].

21.	⊗*Persicaria barbata *(L.) Hara **Polygonaceae **Pirrhe (N). Syn. *Polygonum barbata *Linn.	Stem juice is useful for boils and pimples.	Root paste is applied on the scabies, wounds and swollen parts [28,30].	Stem decoction is useful for ulcers [147].	--	Leaves are astringent, rubifacient and vermifuge [183]. Plant decoction is used to relieve pain and rheumatism [184].

22.	♥•*Bauhinia variegata *L. **Fabaceae **Mountain ebony (E), Koiralo (N), Kachnar, Kovidarah (S). Syn. *B. candida *Ait.	Flower and floral buds are eaten regularly to cure leucorrhoea and mumps.	Flower juice is taken for dysentery and diarrhea [30]. Dried flowers are given for diarrhea, dysentery and piles [31]. Fresh flowers are used as laxative [32].	Flowers are astringent and used for diarrhea and hemorrhage [21].	Butein, hentriacontane, lupeol, nicotiflorin, octacosanol, rhamnopyranoside.	Methanol extract of *B. variegata *bark showed the most remarkable activity as antimicrobial [185] and anticancer [186].

23.	*Ficus religiosa *Linn. **Moraceae **Peepal tree (E), Pipal (N), Aswatha (S).	Bark juice is applied for paralysis.	Bark is astringent, and its decoction is given for gonorrhoea and skin disease [30,31].	Bark is astringent, and used for hemorrhage and healing external wounds [21].	Phytosterolin, vitamin K, tannins.	Methanolic extract of stem bark is useful for memory longevity [187] and used as an analgesic [188]. Phytosterolin is CNS stimulant and hypoglycemic [189].

24.	•⊗*Equisetum diffusum *D. Don **Equisetaceae **Spreading horsetail (E), Ankhle jhar (L), Kurkure (N), 0555/00.	Plant stem juice is given for gonorrhea.	Plant root juice is given for urinary troubles [30], sprains, fractures, burns and scabies [33].	Plant is diuretic and useful for gonorrhea [147].	Apigenin, ascorbic acid, equisetolic acid, folic acid, kaemferol, niacin, silic acid [101,190].	Methanolic plant extract shows good free radical scavenging activity [191].

25.	♥⊗*Parnassia nubicola *Wall. **Parnassiaceae **Mamira (N), 205/00.	Root paste is applied for eye inflammation.	Root paste is useful for wounds [30], body ache, headache, and eye problems [15,33].	--	--	Methanolic root extract showed moderate anti-inflammatory effect [192].

26.	•⊗*Myrica esculenta *Buch.-Ham. ex D.Don **Myricaceae **Box myrtle Bay berry, (E), Kafal (N), Kumbhi, Kaidaryama (S), 567/00. Syn. *M. fraquhariana *Wall.	Fruits are eaten for dysentery and bark decoction is given for bronchitis.	Bark is useful for cough, asthma, sinusitis [31] and chronic bronchitis, diarrhea and dysentery [32].	Bark decoction is useful for asthma, dysentery and lung affections [147].	Friedelin, myricanone, myricadiol, myricanol, myricitrin, taraxerol [181].	Methanolic root extract showed potent anti-inflammatory effect [193].

27.	*Arisaema flavum *(Forsk.) Schott **Araceae **Banko (N), 562/00.	Rhizome juice is applied on earache and skin diseases. Young shoots are cooked as vegetable.	Leaves are consumed as a laxative [15]. Tubers are used for toothache, stomachache and chest infection [29].	--	Alanine, ariseminone, asparagine, cysteine, glycine, norvaline, ornithine [100].	Methanolic tuber extracts revealed weak antiviral property [194].

28.	♥*•⊗Azadirachta indica *A. Juss. **Meliaceae **Neem tree, Margosa tree (E), Neem (N), Aristha, Nimbah (S). Syn. *Melia azadirachta *L.	Both raw and dried leaves are used for fever and blood disorders (no other information given).	Leaves are anthelmintic and good for cough, asthma, piles and urinary discharge [31]. They are used for malarial and intermittent fever, liver complaint and diabetes [32].	Leaves are used for skin diseases and blood circulatory defects [21] and useful for ulcers, sores, swellings and wounds [25].	Azadirachtin, gedunin, limonoids, linoleic acid, nimbin, nimbidin, oleic acid, stearic acid [195].	Nimbidin possesses anti-inflammatory [170], analgesic [196], antipyretic [49], antiulcer, anticholinergic, antihistaminic and antinicotinic effects [197]. Bark extract is useful as antibacterial [198] and antisplasmodial [199]. Leaf extract promotes wound healing, ulcer protective [200] and hypoglycaemic [201].

29.	♥•⊗*Anisomeles indica *(L.) Kuntze **Lamiaceae **Malabar catmint (E), Ratocharpate (N), 167/00. Syn. *A. ovata *R.Br.	Leaf extract is useful for urinary complaints (no other information given).	Plant is astringent, tonic and its juice is useful for urinary affections [30,33].	Plant is taken for uterine affections [147].	Alanine, anisomelic acid, apigenin, amyrin, β sitosterol, behemic acid, betulin, cerotic acid, malabaric acid, ovatodiolide, pedallitin, stearic acid, stigmasterol [27,181].	Ovatodiolide and pedallitin of Anisomeles indica is good anti-inflammatory [202]. Pre-flowering plant water extract is analgesic [203]. Ethanolic leaf extract is strong antiviral [204] and anti HIV potential [205].

30.	♥⊗Lichen species **Lichen **Lichen (E), Jhyau (N), KU 07267.	Lichen extract and decoction is applied to treat moles.	Paste is used as ointment and antibiotic for cuts and wounds [31].	Lichen is cardiac tonic [147].	Atranorin, barbatic acid, norstictic acid, usnic acid, vulpinic acid [112].	*Parmelia species *are antimicrobial and used to treat warts [118,119] and cranial diseases [206].

31.	•⊗*Abies spectabilis *(D.Don) Mirb. **Pinaceae **Himalayan Silver Fir (E), Gobre Salla (L,N), Talispatra (N,S). Syn. *Pinus tinctoria *Wallich ex D. Don	Leaves are sniffed for cough and cold.	Plant needle oil is valued for colds and nasal congestions [30]. Leaf decoction is used for cough and bronchitis [32].	Plant is considered to be used for asthma, bronchitis, cough, rheumatism, anorexia, abdominal lump, indigestion and tuberculosis [22].	β pinene, camphene, carvone, catechin, catechutannic acid, ephedrine, taxine, taxinine[24,32].	Pinene of *Abies *leaves is anti-inflammatory and antidepressant [207]. Plant extract with the ephedrine should always be used with caution in patients with hypertension [38,208].

32.	♥⊗*Quercus lanata *Sm. **Fagaceae **Wooly oak (E), Latyaz (L), Baanjh (N). Syn. *Q. lanuginosa *D.Don	Heart wood is taken as tea and it is laxative in nature.	Resin is useful for soothing body ache [30]. Dry resin is taken to treat dysentery [33].	--	Cyclobalanone, friedelin, pelagonodin, sitosterol, tannins [100].	Resin and bark tannin is anti-inflammatory [122,209].

33.	*Solena heterophylla *Lour. **Cucurbitaceae **Ban kankri (N) KU 07255. Syn. *Melothria heterophylla *L.	Fruits are eaten for common cold and pneumonia of child.	Fruits are useful for throat pain and fever [28].	Root juice is useful for dysuria and spermatorrhoea [147].	Behemic acid, columbin, lignoceric acid [210].	Plant extract is hepato-protective and plant coumarin and flavonoids inhibit platelet aggregation [211].

34.	⊗*Osmanthes fragrans *Lour. **Oleaceae **Tree Jasmine (E), Siringe (N), KU 07244. Syn. *O. acuminatus *(Wall.) Nakai	Leaf juice is taken for fever and cold.	Stem bark is valued for boils, cough and retinitis [30,33].	Leaf juice is tonic [147].	Caffeic acid, catechin, gallic acid, leuropin, ligustroside, luteolin, oleanolic acid, phillyrin, succinic acid [100].	Plant extract has antioxidant and melanogenesis inhibitory effects [212,213] and neuroprotective property [214].

35.	♥⊗*Fragaria nubicola *Lindl. **Rosaceae **Alpine strawberry (E), Bhuikafal (N), KU 07242. Syn. *F. vesca *L.	Fruit paste heals skin diseases and wounds.	Plant juice is useful for inflammation of the nerves and lungs [29]. Root juice is taken for fever [33].	Fruits are astringent and diuretic [147].	Carotenoids, ellagic acid, flavonoids [215].	Ellagic acid of the plant is responsible for antioxidant activity [128]. Plant extract is antimicrobial and anti-inflammatory [101,131].

36.	♥*Curcuma angustifolia *Roxb. **Zingiberaceae **Zeodory, Turmeric (E), Sathi, Kachur (L), Haldi (N) Ban haldi, Haridra, Harita (S) KU 07259. Syn. *C. longa *L.	Rhizome paste is externally applied for paralysis.	Rhizome paste is externally applied to bruises, pains and injuries [31].	Tuber is used for skin diseases and urinary complaints [21]. Fresh tuber juice is antiparasitic and useful for skin affections [25].	Anthraquinone, borneol, campesterol, camphene, caryoplhylene, cineole, curcumin curdione, curzerenone, curlone, eugenol, limonene, linalool, terpinene [100,210].	Curcumin is anti-inflammatory [78-80], antiviral [82], antifungal [83], antispasmodic [86] and hepato-protective [87]. It is also useful for AIDS [90,91] control blood pressure [93]. Plant extract is antimutagenic [216].

37.	*•Evolvulus alsinoides *(L.) Linn. **Fabaceae **Aankuri phul (N), Visnukravita (S).	Decoction of plant is taken for increase memory.	Ash of the plant is spread on boils and pimples [30]. Plant paste is applied on scorpion sting, burns and scabies [33].	Plant is brain stimulant, aphrodisiac, anthelmintic and antidysenteric [217].	β sitosterol, betaine, evolvine, linoleic acid, oleic acid, stearic acid [181].	Plant extract is analgesic, CNS depressant [218] and has anthelmintic, wound healing [219,220] and antibacterial properties [221].

38.	*Sterculia villosa Roxb*. **Sterculiaceae **Sterculia, Odaal tree (E), Odaal (N). Syn. *Firmiana fulgens *(Wall. Ex Master) Corner	Stem bark is considered as an astringent. It is used for cooking breads.	White exudes of the tree is used for throat infection. Root infusion is taken as food adjunct [33].	--	--	Plant extract is useful for skin disease [222].

39.	*⊗Pyracantha crenulata *(D. Don) M. Roem. **Rosaceae **Nepali white thorn (E), Ghangaru (N).	Fruits are eaten for dysentery.	Fruit powder is used for blood dysentery [30,33].	--	Pyracrenic acid, sorbitol, tannin [223].	Pyracrenic acid is anti-inflammatory [223].

40.	*♥⊗Phytolacca acinosa ***Phytolaccaceae **Pokeberry (E), Jaringo (N). Syn. *P. latbenia *(Moq.) H. Walter	Vegetable is consumed for body ache (no other information given).	Plant is narcotic and purgative in properties [30].	--	Acinosolic acid, jailigonic acid, lectins, oleanolic acid, myricadol, phytolaccagenin, spergulagenic acid, zonarol [32,100].	Root extract shows weak triosinase inhibitory activity i.e. Skin whitening [224]. Saponin extracts from *Phytolacca *demonstrated anti-inflammatory [225], antifungal [226] and anti-viral effects [227].

41.	*Smilax aspera *Wall. **Smilacaceae **Rough birdweed (E), Chopchini (L), Kukurdaina (N), 101/00. Syn. *S. capitata *Buch.-Ham. ex D.Don	Root decoction is used for venereal disease.	Root extract cures scabies [30] and purifies blood [33].	--	Asparagenin, engelitin, parallin, pseudogenin, rutinoside, sarsapogenin, smilogenin, tannin [126,228].	Stem juice is used for dropsy and gout [229]. Rutinoside is cancer inhibitory [230].

42.	*♥⊗Ficus auriculata *Lour. **Moraceae **Eve's apron (E), Timila (N). Syn. *F. roxburghii *Wall	Stem juice is considered effective against diarrhea and fruits are consumed for dysentery.	Bark juice and roasted figs are useful for diarrhea and dysentery [28,30].	--	β sitosterol, epifriedelanol, friedelin [100].	Tannins of the bark extract may reveal anti-inflammatory and analgesic activities [231].

43.	*♥•⊗Euphorbia hirta *Linn. **Euphorbiaceae **Snake weed, Asthma weed (E), Dudhi jhar (N), Pusitoba (S). Syn. *E. pilulifera *L.	Plant latex is applied for cuts. Plant juice is applied in asthma and diarrhea.	Plant juice is useful for boils, cuts and wounds [30] and is considered to be used in treatment of asthma and cough [32].	It is useful for cardiovascular complaints, asthma and spleen disorders [27].	Galloylquinic acid, Phorbol acid, leucocyanidol, quercitol, camphol, quercetin, chlorophenolic acid, shikimic acid [100].	Plant alkaloid is effective in broncho-dilation [27], and used as an antispasmodic, antiasthmatic, expectorant, anticatarrhal [74,232]. The methanol extract of flowers has antibacterial activity [75].

44.	*⊗Jurinea dolomiea *Bioss. **Asteraceae **Bhutkes (N) KU 07266. Syn. *Carduus macrocephalus *Wall.	Root decoction is taken in stomachache and diarrhea.	Root is used for stomachache and diarrhea [29]. Root juice is taken for cough and cold [30].	--	Vasicine.	Vasicine exhibited strong respiratory stimulant, moderate hypotensive, cardiac-depressant and abortifacient [233].

45.	*♥•⊗Tinospora sinensis *(Lour.) Merr. **Menispermaceae **Heart leaved Moonseed (E), Gurjo (N), Guduchi, Amritavali (S). Syn. *T. cordifolia *auct. non L.	Dilute stem juice is drunk for diabetes.	Stem juice is valued for dysentery, diabetes, gonorrhoea [31], genital disorders and diabetes [32].	Stem is used for urinary diseases and hepatitis [21]. Stem juice is antipyretic, antiperiodic and alterative [25].	Berberine, choline, cordifol, isocolumbin, jatrorhizine, magnoflorine, palmatine, tembeterine, tinosporin, tinosporide [27].	Water extract (berberine) is antipyretic [234] and antidiabetic [66] due to berberine [68] but higher doses may be antagonistic [69]. Plant extract is hepato-protective [235], hypoglycaemic [67] and immunostimulant [236].

46.	♥⊗*Betula utilis *D.Don **Betulaceae **Himalayan Birch (E), Bhuj pat (L), Bhojpatra (N), Bhurjah, Lekhyapatrak (S), 556/00. Syn. *B. bhojpattra *Lindl.	Bark decoction is useful for sore throat.	Bark is used for bacterial infections, skin diseases, bronchitis cough [15,33], and cuts, wounds and burns [30].	Bark is astringent and its fume is used for easy delivery and placenta expels [21].	Betulin, karachic acid, leucocyanidin, lupenone, lupeol, oleanolic acid.	Bark extract is antiseptic [100]. Betulinic acid is anti-inflammatory [237].

47.	*♥•⊗Aesculus indica *(Colebr. ex Cambess.) Hook. **Hippocastanaceae **Horse chesnut (E), Panger, Karu (N), Naaru (S), Horse 563/00.	Seed oil is valued for joint pain and skin problems (no other information given).	Seed oil is used for scabies and skin diseases [15,30,33].	Bark is used for dislocated joints and seed oil is considered to be used for rheumatism [147].	Aescin, aesculuside, astragalin, β sitosterol, catechol, decanoic acid, epicatechin, quercetin, rutin, saponins [100].	Plant is used for delaying hypersensitivity [238]. Aescin is cardio-stimulant and anti-inflammatory [239].

48.	♥⊗*Daphne bholua *Buch.-Ham. ex D.Don **Thymelaeaceae **Nepali paper plant (E), Gore, Baruwa (L), Lokta, Kagaj pate (N). Syn. *D. cannabina *Lour. ex Wall.	Seeds are taken for stomachache and anthelmintic.	Root extract is used for intestinal disorder and powered seeds are taken as an anthelmintic [28].	--	Daphnoside, daphnetin, genkwanin, luteolin, taraxerol [100].	Bark decoction is given to treat fever. Root juice is anthelmintic [240].

E = English, L = Local, N = Nepali, S = Sanskrit, Syn. = Synonymous

*⊗ *= Species's use resembled with the common uses of *Ayurveda, • *= Species's use resembled with earlier reports, ♥ = Species's use resembled with latest common phytochemical findings

### Pharmacology

The results obtained support prior observations, pharmacology and Ayurvedic uses concerning the following species: the crude extracts of *Acacia catechu *for cold and cough, *Aconitum spicatum *as analgesic, *Aesculus indica *for joint pain, *Andrographis paniculata *for fever, *Anisomeles indica *for urinary affections, *Azadirachta indica *for fever, *Euphorbia hirta *for asthma, *Taxus wallichiana *for tumor control, and *Tinospora sinensis *for diabetes. This probably explains the use of these plants by indigenous people against a number of infections as transcend from transcultural environment with following home herbal remedy, *Ayurveda *and *Baidhya *systems. It is known that the families Rutaceae and Meliaceae are among the richest and most diverse sources of secondary metabolites among the angiosperms [44], and the species of Meliaceae are known to have intense antimalarial characters due to highly oxygenated terpenoids [45]. Use of leaves of *Azadirachta indica *(Meliaceae) as antipyretic is widely used in study area (Table 1) and throughout Nepal [46] was substantiated by the nimbidin flavonoids [47,48]. Oleic acid and gedunin of *A. indica *are also reported to be an *in vitro *antimalarial [49-51]. Other species contributed as antipyretic in home herbal remedy in study area were *Andrographis paniculata *(Acanthaceae), *Aconitum spicatum *(Ranunculaceae) and *Osmanthes fragrans *(Oleaceae).

Andrographolide and neoandrographolide from *Andrographis paniculata *own anti-inflammatory activity [52,53]. Its diterpene exhibits antioxidant and hepato-protective properties [54-57]. Immunostimulant [58], antibacterial [59], analgesic [60] and antiprotozoal [61] characteristics of *A. paniculata *extract have also been demonstrated. These values probably explain the use of *A. paniculata *by the indigenous people against a number of infections and fever. Crude root extract of *Podophyllum hexandrum *(Berberidaceae) was used as hepato-protective, despite the hepatotoxic character reported due to its lignans [62]. Podophyllotoxin has manifested antimitotic activity and capability of inhibiting DNA, RNA and protein synthesis [63]. There were seven species in study area exhibiting hepato-protective effects. Among them, six were pharmacology based and three were folkloric. Plant extracts of *P. hexandrum *and *Andrographis paniculata *showed hepato-protective characters consistent with the folk use and pharmacology.

Alkaloids are most common in flowering plants, especially in Fabaceae, Ranunculaceae and Solanaceae [64]. Some alkaloids (aconitine, anisodamine, berberine, charantine, leurosine) show antidiabetic effects [65]. Berberine of *Tinospora sinensis *(Menispermaceae) is antidiabetic [66-68], but higher doses may be antagonistic [69], which strongly support the folkloric use of the plant extract. According to Marles and Farnsworth [70], there are about 1,000 species of plants that can act as an antidiabetic and approximately 80% of these are used in folk herbal medicine. Antidiabetic reports of *Azadirachta indica, Carum carvi, Tinospora sinensis *and *Vitex negundo *stated in the present communication were pharmacologically rationale and that of *A. indica *and *T. sinensis *was folk-based.

Euphorbiaceae species are generally characterized by milky latex [71], and sticky saps are co-carcinogenic, and can cause severe skin irritation and are toxic to livestock and humans [72]. They are rich in active compounds including terpenoids, alkaloids, phenolics and fatty acids, having ethnopharmaceutical uses [73]. *Sapium insigne *(Euphorbiaceae) is skin irritant, and commonly used as fish poison in study area and throughout Nepal [28]. Both the water and methanol extracts of *Euphorbia hirta *(Euphorbiaceae) are antibacterial [74,75] and effective as expectorant [76,77] and broncho-dilator [27], which is consistent with the folkloric use in treatment of respiratory complaints.

Pharmacologically, curcumin of *Curcuma *species (Zingiberaceae) acts as an anti-inflammatory [78-80], antibacterial [81], antiviral [82], antifungal [83], antitumor [84,85], antispasmodic [86], and hepato-protective [87]. The oxygen radical scavenging activity of curcumin has been implicated in its anti-inflammatory effects [88,89] thus curcumin may prove useful as a drug for arthritis, cancer, HIV [90-92] and high blood pressure [93]. Wide range of pharmacological reports including antibacterial and antiviral complements the folk use to treat paralysis. Rhizome extract of the plant was widely used for skin diseases (bruises, injuries, etc.) in west Nepal [15] and in the *Ayurveda *[21].

The folk use of *Acacia catechu *(Fabaceae) wood tea as an expectorant fairly corroborated the pharmacological properties because the tannin and cyanidanol [94,95] of the plant impart astringent activity which helps to recuperate diarrhea. Tannins are also known as antimicrobial [96] and triterpenoids are beneficial for inflammation and cancer [97]. The hepato-protective and hypoglycemic properties of *A. catechu *could be attributed to the quercetin [98] and epicatechin [99] respectively. Leaf extracts of *Taxus wallichiana *(Taxaceae) inhibit pregnancy in rats [22], vitiate blood disorders [100] and control tumor growth [101]. In the study area, *Taxus *leaf juice is used for treatment of cancer and bronchitis.

Lectins of *Cannabis sativa *(Cannabaceae) possess haema-gluttinating properties [38] which corroborate the indigenous use of the leaf extract to control bleeding. Crude leaf extract of *Vitex negundo *(Verbenaceae) is recommended as antitussive and anti-asthma [102], antibacterial [103], antifungal [104], hypoglycemic [105], anti-cancer [106], acne control [107], inhibitor of edema [108,109] to tracheal contraction [110]. However, it did not corroborate the folk use for stomachache but was partially complemented by earlier observations [30,111]. The unlike uses of the species after thorough scrutiny, under different medical systems and comparisons pose more research scopes. Several instances are rational behind a certain function of a phytomolecule sometimes inconsistent to the pharmacology and ethnopharmacology. Moreover, while advocating herbal medicine as alternative therapy, toxicity of plants should be borne in mind.

Lichens and their metabolites have manifold biological activity: antiviral, antibiotic [112], antitumor, allergenic, plant growth inhibitory, antiherbivore, ecological roles and enzyme inhibitory [113,114]. Usnic acid and vulpunic acid (produced by mycobiont) of lichens are mitotic regulators [115] and own antibiotic properties [116]. *Parmelia sulcata *lichen manifests antibacterial and antifungal activities [117,118]. Use of *Parmelia *species to treat warts [119] is analogous to its folk use. Folk use of wood tea of *Quercus lanata *(Fagaceae) as a laxative may verify the actions of tannin. Tannins reveal activities against central nervous system disorders [120] and inflammation [121,122]. Further pharmacological evaluation of the extracts of those species which reveal weak pharmacological validities are needed before they can be used as therapeutic potentials.

The compounds which contribute to the antioxidative properties are polyphenols [123], vitamin C [124], β carotene [125], anthocyanins [126], and flavonoids [127]. Ellagic acid of *Fragaria nubicola *(Rosaceae) is also responsible for antioxidant activity [128]. Antioxidants are associated with reduced risk of cancer and cardiovascular diseases [129] and many other ailments [130]. Antimicrobial and anti-inflammatory properties of *Fragaria *fruit extracts [101,131] are consistent with the folkloric use as remedy for skin diseases and wounds. The usage of root powder of *Potentilla fulgens *(Rosaceae) as a dentifrice is common in the study area and throughout Nepal [132,133] and it is in accord to the Ayurvedic uses. However, the usage is yet to be verified pharmacologically.

**Figure 2:**
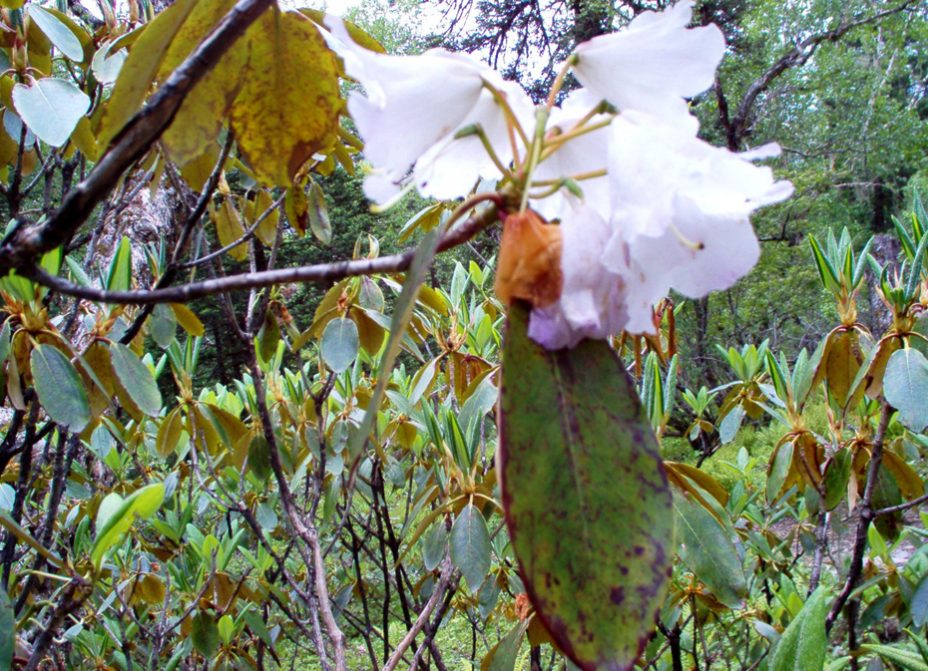
***Rhododendron campanulatum *D.Don (*Chimal*): Flowers are used in body ache and throat pain**.

Wogonin of *Scutellaria discolor *(Lamiaceae) is considered as a most potent antiviral [134] and anxiolytic [135] compound. Plant root extract is also useful for rheumatism [136]. Whole plant and leaf paste is useful for cuts & wounds, which probably rationalize the activities of wogonin. Linalool also possesses an anxiolytic effect [137], and this effect probably substantiates the folk uses of *Skimmia anquetilia *(Rutaceae) leaves as medicine for headache and freshness. Linalool is the main constituent of *Skimmia *root. The indigenous uses of six species *Arisaema flavum, Ficus religiosa, Rhododendron campanulatum *(Figure 2), *Smilax aspera, Solena heterophylla *and *Sterculia villosa *repudiated to any of the comparables, since these uses were additional to the Nepalese ethnomedicinal vault and these addition demands further research.

*Aconitum spicatum *(Ranunculaceae), taken alone is poisonous, so it is never used alone by the local communities. A paste made from its roots is applied as antipyretic and analgesic after mixing with *Terminalia chebula *(Combretaceae). Folk use of root extract of *A. spicatum *as an analgesic is consistent to the anti-inflammatory activity of caffeic acid found in the plant extract [138]. About 80% of plant alkaloids possesses anti-inflammatory properties and among them isoquinoline (berbamine, berberine, cepharanthine and tetrandine) was the most active [139,140]). Diterpenoid alkaloids, commonly isolated from the plants of Ranunculaceae family, are commonly found to have antimicrobial properties [141]. Folk uses as antipyretic and analgesic of *A. spicatum *root extract are validated by the *in vitro *antimicrobial properties. In some cases, multi-component therapy has been practiced and considered as effective as Kareru *et al*. [142] observed in Kenya, but the present assessment considered only the primary one to discuss. We believe that the associate plants must also be considered as excellent candidates for future studies to determine the mechanisms of their activity, as well as for the isolation and identification of active constituents [143,144]. Thus, traditional herbal medicine renders primary health care needs of two thirds of the rural population of the Nepalese, represents a largely unexplored source for potential development of new drugs [145,146].

## Conclusions

Validation of the ethnomedicinal uses of 48 Nepalese medicinal plants using comparative assessment with the common uses of the *Ayurveda*, earlier studies and the latest phytochemical findings showed that the folk uses of only about 50%, 70% and 40% of plant species respectively exhibited affinity. The folk uses of *Acacia catechu *for cold and cough, *Aconitum spicatum *as an analgesic, *Aesculus indica *for joint pain, *Andrographis paniculata *for fever, *Anisomeles indica *for urinary affections, *Azadirachta indica *for fever, *Euphorbia hirta *for asthma, *Taxus wallichiana *for tumor control, and *Tinospora sinensis *for diabetes are consistent with the latest pharmacological findings, as well as common Ayurvedic and earlier uses. However, the frequent folk uses of *Arisaema flavum, Ficus religiosa, Rhododendron campanulatum, Smilax aspera, Solena heterophylla *and *Sterculia villosa *of study area repudiated at all. The preliminary results obtained from the present assessment indicate that further investigation of ethnopharmacology is worthwhile. The validity assessment from the present research provided the potential to identify, research, and use which plants and their ingredients are the most significant for treatment of particular diseases.

## Competing interests

The authors declare that they have no competing interests.

## Authors' contributions

All authors share the contributions to this manuscript. RMK carried out field research, analyzed the data, and wrote the manuscript, and KPS and RWB designed the study, supervised the work, collected the literature, and revised the manuscript. All authors approved the final version of this manuscript.
